# The clinical outcomes of selective and spontaneous fetal reduction of twins to a singleton pregnancy in the first trimester: a retrospective study of 10 years

**DOI:** 10.1186/s12958-022-00935-0

**Published:** 2022-04-22

**Authors:** Chao Wang, Fei Tang, Bing Song, Guanjian Li, Qiong Xing, Yunxia Cao

**Affiliations:** 1 Department of Obstetrics and Gynecology, the First Affiliated Hospital of Anhui Medical University, No 218 Jixi Road, Hefei, 230022 Anhui China; 2 NHC Key Laboratory of Study On Abnormal Gametes and Reproductive Tract (Anhui Medical University), No 81 Meishan Road, Hefei, 230032 Anhui China; 3 Key Laboratory of Population Health Across Life Cycle (Anhui Medical University), Ministry of Education of the People’s Republic of China, No 81 Meishan Road, Hefei, 230032 Anhui China; 4 Anhui Province Key Laboratory of Reproductive Health and Genetics, No 81 Meishan Road, Hefei, 230032 Anhui China; 5 Biopreservation and Artificial Organs, Anhui Provincial Engineering Research Center, Anhui Medical University, No 81 Meishan Road, Hefei, 230032 Anhui China

**Keywords:** Spontaneous fetal reduction, Selective fetal reduction, Singleton pregnancy, Multifetal pregnancies, Pregnancy complications

## Abstract

**Background:**

Singleton pregnancy is encouraged to reduce pregnancy complications. In addition to single embryo transfer (SET), selective and spontaneous fetal reduction (SEFR and SPFR) can also achieve singleton pregnancies. After SEFR or SPFR, an inanimate fetus remains in the uterus. It is unclear whether the inanimate fetus would adversely affect another fetus or the mother. Previous studies have focused on the differences between pre- and post-reduction. However, studies focusing on the influence of SEFR and SPFR on the remaining fetal development and maintenance of pregnancy are rare.

**Methods:**

Materials from 5922 patients whose embryo transfer dates ranged from March 2011 to January 2021 were collected. Both the SEFR group (*n* = 390) and SPFR group (*n* = 865) had double embryos transferred (DET) and got twin pregnancies, but subsequent selective or spontaneous fetal reduction occurred. The SET group (*n* = 4667) had only one embryo transferred. All were singleton pregnancies on the 65th day after embryo transfer. Clinical outcomes, including pregnancy outcomes, pregnancy complications, and newborn outcomes, were compared among the three groups.

**Results:**

After adjusting for age, infertility duration, types of infertility, states of embryos, body mass index, and factors affecting SET or DET decisions, multivariate regression analysis revealed that SEFR increased the risk of miscarriage (OR 2.368, 95% CI 1.423–3.939) and preterm birth (OR 1.515, 95% CI 1.114–2.060), and reduced the gestational age (βeta -0.342, 95% CI -0.544– -0.140). SPFR increased the risk of gestational diabetes mellitus (GDM) (OR 1.657, 95% CI 1.215–2.261), preterm premature rupture of membranes (PPROM) (OR 1.649, 95% CI 1.057–2.574), and abnormal amniotic fluid volume (OR 1.687, 95% CI 1.075–2.648). Both SEFR and SPFR were associated with reduced live birth rate (OR 0.522, 95% CI 0.330–0.825; OR 0.671, 95% CI 0.459–0.981), newborn birth weight (βeta -177.412, 95% CI -235.115–-119.709; βeta -42.165, 95% CI -83.104–-1.226) as well as an increased risk of low-birth-weight newborns (OR 2.222, 95% CI 1.490–3.313; OR 1.510, 95% CI 1.092–2.087).

**Conclusions:**

DET with subsequent fetal reduction was related to poor clinical outcomes. We recommend that DET with subsequent fetal reduction should only be considered as a rescue method for multiple pregnancy patients with potential complications, and SET is more advisable.

## Introduction

Multifetal pregnancy is prevalent in assisted reproduction technology (ART), which can result in a series of complications such as gestational diabetes, hypertensive disorders, premature rupture of membranes, premature birth, etc. [1–4]. Although the incidence rate of multifetal pregnancy differs among all reproductive centers worldwide, ART has contributed to a substantial proportion of all twins, triplets, and higher-order infants born [5]. To decrease the incidence of multifetal pregnancies, single embryo transfer (SET) has received increasing attention in recent years. However, in a large number of cases, more than one embryo is still transferred into the uterus due to patient requests or the pursuit of a successful pregnancy in one transfer cycle by reproductive centers or doctors. The rate of multifetal pregnancies remains high. In 2018, the European Society of Human Reproduction and Embryology (ESHRE) found that the proportions of singleton, twin, and triplet deliveries after in vitro fertilization (IVF)/ intracytoplasmic sperm injection (ICSI) were 82.5%, 17.5%, and 0.5%, respectively [6]. To reduce the risk of complications in multifetal pregnancies, a technology known as selective fetal reduction (SEFR), was developed [7]. This is a method to reduce the number of fetuses through surgery. Moreover, the incidence of spontaneous fetal reduction (SPFR) can also reduce multifetal pregnancies to singleton pregnancies. Luo reported that the incidence of SPFR was 17.1% in dichorionic diamniotic twin pregnancies [8]. Unfortunately, its occurrence is difficult to predict based on existing research. Regardless of the method, double embryo transfer (DET) with subsequent selective and spontaneous fetal reduction can achieve singleton pregnancy status, but in either method, an inanimate fetus remains in the uterus. It is unclear whether an inanimate fetus would adversely affect another fetus or the mother. Therefore, it is unclear whether DET with subsequent SEFR or SPFR should replace SET. Research on these issues is rare. The current study collected a large amount of data and compared the clinical outcomes, including pregnancy outcomes, pregnancy complications, and newborn outcomes, of singleton pregnancies from SEFR, SPFR, and SET.

## Methods

### Patients

Materials from 5922 patients whose embryo transfer dates ranged from March 2011 to January 2021 in our center were analyzed. Both the SEFR group (*n* = 390) and SPFR group (*n* = 865) had DET and got dichorionic twin pregnancies, but subsequent selective or spontaneous fetal reduction occurred. The SET group (*n* = 4667) had only one embryo transferred. All were singleton pregnancies on the 65th day after embryo transfer (equivalent to 12 weeks of gestational age). The study was approved by the Research Ethics Board of the hospital.

### Treatments and follow-up

All transferred embryos were at the blastocyst stage. For the fresh embryo transfer cycles, one or two embryos were transferred on the fifth day after oocyte retrieval. For the frozen embryo transfer cycles, at least two menstrual cycles later, one or two frozen-thawed embryos were transferred. SET is the first choice for specific patients (i. first transfer cycle, ii. scarred uterus, iii. age < 38 years with a child already, iv. BMI ≤ 18 kg/m2 or BMI ≥ 30 kg/m2, v. müllerian anomalies, vi. history of twin pregnancy miscarriage, vii. cervical troubles, viii. uterine fibroids, or ix. personal preference for SET). Trans-vaginal ultrasound (TVS) was conducted between the 25th- and 30th day after transfer, and it was considered to be a clinical pregnancy if a gestational sac could be found in the uterus. In this study, patients in the SPFR and SEFR groups both had two gestational sacs during the first TVS examination. As the name suggests, the SET group only received one embryo transfer, and only one gestational sac was seen. The SEFR operation was executed between the 30th- and 65th day after transfer. SPFR referred to the spontaneous loss of cardiac activity of one fetus.

Information in the first trimester, such as patient characteristics, singleton or twin pregnancy, time of fetal reduction, etc., was obtained from the patient's primary medical records. Information in the second and third trimesters, such as pregnancy complications, neonatal outcomes, etc., was obtained by telephone follow-up. All information was input into the clinical database, and we downloaded the required data from the database for this research.

### SEFR

All patients were informed of the risks associated with this operation and signed informed consent forms. After disinfection of the vulva, vagina, and cervix, TVS was used to evaluate the sac site, sac size, fetal length, and yolk sac diameter. A gestational sac with a smaller size, shorter fetal length, bigger yolk sac, or relatively closer to the cervix (if no differences were found in the first three items) was selected as the target sac. The COOK 17G needle (K-OSN-1735-B-90, COOK, Australia) was inserted transvaginally into the site where the pulsating heart was beating and then aspirated repeatedly until the loss of heart beats with a negative pressure of 300 mmHg. This process was monitored using the TVS.

### Statistical analyses

All statistical analyses were performed using SPSS 22.0. Continuous data was compared with a one-way ANOVA test and presented as mean ± standard deviation, and the SNK test was used for comparison between two groups. Enumeration data was compared using the chi-square test and presented as %(n). Multivariate regression analysis was used to derive odds ratio (OR) estimates for the association of singleton pregnancies from selective and spontaneous fetal reduction and single embryo transfer with pregnancy outcomes, pregnancy complications, and newborn outcomes. The confounders considered in the current study were age, infertility duration, types of infertility (primary or secondary), states of embryos (fresh or frozen), body mass index (BMI), and factors affecting SET or DET decisions. The odds ratio (OR)/ βeta and 95% confidence interval (CI) were calculated. *P*-value (2-tailed) < 0.05 was considered statistically significant.

## Results

### Patient characteristics

The study was comprised of 5922 pregnant women. The basic characteristics of the three groups are described in Table 1. Age, infertility duration, types of infertility, states of embryos, BMI, time of fetal reduction, and partial factors affecting SET or DET decisions were significantly different among the three groups.

**Table 1:** Patient characteristics (*X* ± *S*, %(n))

	SEFR (*n* = 390)	SPFR (*n* = 865)	SET (*n* = 4667)	*F/X*^*2*^*/t*	*P*
Age (years)	31.14 ± 3.90^ab^	30.61 ± 4.39^c^	29.60 ± 4.34	38.63	< 0.001
Infertility duration (years)	3.47 ± 2.60^a^	3.83 ± 2.77^c^	3.36 ± 2.53	11.86	< 0.001
Types of infertility				9.12	0.010
Primary infertility	54.87(214)	61.38(531)^c^	56.03(2615)		
Secondary infertility	45.13(176)	38.61(334)^c^	43.97(2052)		
States of embryos				16.45	< 0.001
Fresh embryos	17.18(67)^b^	15.61(135)^c^	11.87(554)		
Frozen embryos	82.82(323)^b^	84.39(730)^c^	88.13(4113)		
BMI (kg/m^2^)	21.58 ± 2.96^ab^	22.28 ± 3.09	22.33 ± 3.11	10.34	< 0.001
Time of fetal reduction (days)^*^	42.24 ± 4.83	37.27 ± 8.35	-	13.53	< 0.001
Factors affecting SET or DET decisions					
Scar uterus	15.90(62)^a^	3.24(28)^c^	15.11(705)	90.65	< 0.001
Müllerian anomalies	2.05(8)	1.16(10)	1.31(61)	1.76	0.420
Cervical troubles	1.28(5)	0.23(2)	0.66(31)	4.83	0.076
Uterine fibroids	5.90(23)^ab^	1.73(15)	1.52(71)	38.21	< 0.001

*SEFR* Singleton from selective fetal reduction, *SPFR* Singleton from spontaneous fetal reduction, *SET* Single embryo transfer, *DET* Double embryos transfer, *BMI* Body mass index, ^*^ indicates the time interval between the fetal reduction and embryo transfer ^a^ indicates SEFR and SPFR have significant difference; ^b^ indicates SEFR and SET have significant difference; ^c^ indicates SPFR and SET have significant difference

### Pregnancy outcomes and complications

As shown in Table 2, the rates of miscarriage, live birth, GDM, and PPROM were significantly different among the three groups. Compared to SET, SEFR had a higher miscarriage rate and lower live birth rate, and SPFR had a higher rate of GDM and PPROM. There were no statistically significant differences in the rates of gestational hypertension, placental abnormalities, and abnormal amniotic fluid volume among the three groups.

**Table 2:** Pregnancy outcomes and complications (% (n))

	SEFR (*n* = 390)	SPFR (*n* = 865)	SET (*n* = 4667)	*X*^*2*^	*P*
Miscarriage	5.13(20)^b^	2.54(22)	2.08(97)	14.78	0.001
Live birth	93.85(366)^b^	95.61(827)	96.91(4523)	12.62	0.002
GDM	6.15(24)	6.94(60)^c^	4.37(204)	24.05	< 0.001
Gestational hypertension	7.95(31)	7.63(66)	6.73(314)	1.58	0.455
Placental abnormalities	5.90(23)	5.90(51)	6.71(313)	1.06	0.588
PPROM	2.82(11)	3.35(29)^c^	2.01(94)	6.50	0.039
Abnormal amniotic fluid volume	2.05(8)	3.12(27)	1.95(91)	4.82	0.090

*GDM* Gestational diabetes mellitus, *PPROM* Preterm premature rupture of membranes, ^a^ indicates SEFR and SPFR have significant difference, ^b^ indicates SEFR and SET have significant difference, ^c^ indicates SPFR and SET have significant difference

### Newborn outcomes

The newborn outcomes are described in Table 3. There were 367 newborns in the SEFR group, 829 in the SPFR group, and 4529 in the SET group. No statistically significant differences were found in the birth defect rate. However, newborns in the SEFR group had the shortest gestational length and lowest birth-weight. SEFR and SPFR both had a higher rate of low-birth-weight newborns than SET.

**Table 3:** Newborn outcomes (*X* ± *S*, %(n))

	SEFR (390)	SPFR (865)	SET (4667)	*F/X*^*2*^	*P*
Delivery cases	367	829	4529	-	-
Gestational week	38.39 ± 2.08^ab^	38.59 ± 1.96	38.71 ± 1.84	5.79	0.003
Preterm birth	14.99(55)	13.03(108)	11.08(502)	6.92	0.031
Newborn birth weight (g)	3191.62 ± 568.70^ab^	3342.07 ± 566.44	3391.75 ± 528.00	25.11	< 0.001
Low-birth-weight newborns	8.72(32)^b^	6.63(55)^c^	4.28(194)	20.50	< 0.001
Birth defect	1.91(7)	2.17(18)	1.83(83)	0.44	0.804

^a^ indicates SEFR and SPFR have significant difference; ^b^ indicates SEFR and SET have significant difference; ^c^ indicates SPFR and SET have significant difference

### Multivariate regression analysis on clinical outcomes

Multivariate regression analysis was conducted to analyze the effect of different groups on pregnancy outcomes, pregnancy complications, and newborn outcomes after adjusting for age, infertility duration, types of infertility (primary or secondary), states of embryos (fresh or frozen), BMI, and factors affecting SET or DET decisions. Compared to SET, SEFR increased the risk of miscarriage (OR 2.368, 95% CI 1.423–3.939) and preterm birth (OR 1.515, 95% CI 1.114–2.060) and reduced the gestational age (βeta -0.342, 95% CI -0.544– -0.140). SPFR increased the risk of gestational diabetes mellitus (GDM) (OR 1.657, 95% CI 1.215–2.261), preterm premature rupture of membranes (PPROM) (OR 1.649, 95% CI 1.057–2.574), and abnormal amniotic fluid volume (OR 1.687, 95% CI 1.075–2.648). Both SEFR and SPFR were associated with reduced live birth rate (OR 0.522, 95% CI 0.330–0.825; OR 0.671, 95% CI 0.459–0.981) and newborn birth weight (βeta -177.412, 95% CI -235.115–-119.709; βeta -42.165, 95% CI -83.104–-1.226), as well as an increased risk of low-birth-weight newborns (OR 2.222, 95% CI 1.490–3.313; OR 1.510, 95% CI 1.092–2.087) (Table 4).

**Table 4:** Multivariate regression analysis for clinical outcomes (*OR*/*βeta* (95% *CI*))

	SEFR	SPFR	SET
Miscarriage	**2.368(1.423, 3.939)**	1.238(0.763, 2.009)	ref
Live birth	**0.522(0.330, 0.825))**	**0.671(0.459, 0.981)**	ref
GDM	1.329(0.844, 2.095)	**1.657(1.215, 2.261)**	ref
Gestational hypertension	1.238(0.833, 1.842)	1.120(0.840, 1.493))	ref
Placental abnormalities	0.847(0.544, 1.318)	0.972(0.711, 1.329)	ref
PPROM	1.712(0.898, 3.264)	**1.649(1.057, 2.574)**	ref
Abnormal amniotic fluid volume	1.056(0.503, 2.217)	**1.687(1.075, 2.648)**	ref
Gestational age	**-0.342(-0.544, -0.140)**	**-0.155(-0.298, -0.012)**	ref
Preterm birth	**1.515(1.114, 2.060)**	1.230(0.976, 1.550)	ref
Newborn birth weight	**-177.412(-235.115, -119.709)**	**-42.165(-83.104, -1.226)**	ref
Low-birth-weight newborns	**2.222(1.490, 3.313)**	**1.510(1.092, 2.087)**	ref
Birth defect	1.076(0.490, 2.361)	1.222(0.721, 2.072)	ref

Adjusted for age, infertility durations, types of infertility, states of embryos, BMI and factors affecting SET or DET decisions; *OR* for enumeration data and *βeta* for Continuous data; Bold values indicate statistical significance

## Discussion

This study collected a large amount of data over 10 years. We compared the obstetric and neonatal outcomes of 5922 pregnancies, including 4667 singletons following SET, and twins following DET and subsequently reduced either spontaneously (SPFR, 865 cases) or surgically (SEFR, 390 cases). We found that SET resulted in an overall better outcome than either SPFR or SEFR, with lower risk of several pregnancy complications as well as better results for gestational age and birth-weight.

There is no dispute that twin or higher-order pregnancies can result in many complications. Thus, an increasing number of reproductive centers have begun to encourage SETs. While SETs have decreased the multifetal pregnancy rate, the rate is still higher than that in natural conception, whether in Europe, America, or China [5, 6, 9]. In our center, SET is the first choice for specific patients, as listed in the Method section. Similar to the population seen at our center, patients who do not meet these conditions comprise a large proportion of all patients. In these populations, SET is thought to decrease the incidence of complications. However, the success of ART treatment—that is, a successful pregnancy —is also an important target and should be taken into consideration in the protocol selection process. One meta-analysis reported that the clinical pregnancy rate may be lower in women who had SETs than in those who had DET, and the live birth rate may also be reduced in those with SET [10]. Furthermore, repeated treatment, advancing age and urgency to become pregnant are factors that moderate a woman's choice of SET, ignoring the risks of multifetal pregnancies [11]. In addition, some patients were seeking twins and wished to minimize physical and psychological stress by having as few IVF treatments as possible [12]. Of course, many physicians or centers did not offer SET due to their pursuit of a successful rate in one transfer cycle [11, 13]. For doctors or patients, weighing the safety and success rate of assisted reproductive technology may be a difficult task. These factors explain why DET is still popular.

For pregnant women who are not suitable or are unwilling to have a twin pregnancy, in addition to SET, singleton pregnancy can be achieved in two ways: waiting for SPFR or performing surgical SEFR if a multifetal pregnancy occurs. The SPFR rate in dichorionic diamniotic twin pregnancies was approximately 17.1%, with most cases occurring in the first trimester [8]. In all multifetal pregnancies, the rate was 38% [14]. Limited studies have reported that the risk factors for SPFR may be related to age, endometrial thickness on the day of hCG administration, and the initial number of gestational sacs [15–17]. Despite these studies, SPFR remains largely unpredictable. Waiting for the occurrence of SPFR blindly will delay the SEFR procedure which may lead to increased risks from SEFR surgery [18, 19]. Surgical SEFR technology is more flexible, as doctors can decide when to conduct it and which fetus should be reduced [20]. In terms of the technology itself, SEFR has already been proven to have a success rate of almost 100% [21].

Many previous studies have demonstrated that SPFR and SEFR had better or comparable perinatal outcomes than those in the non-reduced multifetal pregnancy group [8, 22–24]. However, only a few studies have focused on the outcomes of twin pregnancies reduced by SPFR/SEFR compared to original singleton pregnancies. In the current study, compared to original singleton pregnancies, SPFR led to a similar miscarriage rate but a poor live birth rate and newborn birth weight. In a study of dichorionic diamniotic twin pregnancies, Luo further reported that the live birth rate, take-home baby rate, neonatal birth weight, and other primary outcome measures in the singleton after SPFR were not inferior to those in cases of original singleton pregnancies [8]. Luo’s work included pregnancies where SPFR occurred ≤ 7 weeks of gestational age (42.1%), 8–12 weeks (47.1%) and 12–27 weeks (9.4%); by contrast, our study only included pregnancies where SPFR occurred within 12 weeks of gestational age. Although, the time of fetal reduction was divided into different groups by Luo, no further analysis of the differences in outcomes between the groups was carried out. Pinborg et al. believed that SPFR occurring at > 8 weeks of gestational age was related to a higher risk of adverse obstetric outcomes in IVF singletons [25]. It should be noted that, in all studies, for SPFR, there may be an interval between the recorded fetal reduction time and the real fetal reduction time, due to the patients’ delayed examination. Further, the singleton pregnancies in the current study were from SET, but in Luo’s and Pinborg’s studies, they may be from SET, DET, or higher embryos transfer. It has been revealed that the preterm birth rate, extremely preterm birth rate, low-birth weight rate and very low-birth weight rate of singleton pregnancies increased with the increase of embryo transfer numbers [26]. A similar conclusion was reached by Poikkeus’s study [27]. As for SEFR, Cheang et al. indicated that twin pregnancies from SEFR had a higher incidence of extreme-prematurity, prematurity, and lower birth weight than twin pregnancies without fetal reduction [28]. Similarly, SEFR in this study also reduced the length of gestational age and newborn birth weight. Meanwhile, SEFR increased the miscarriage rate and reduced the live birth rate compared to those with original singleton pregnancies. It is difficult to find literatures focusing on the differences in pregnancy complications between the original singleton group and the SPFR/SEFR group. Our research provides evidence that SPFR increases the rate of GDM, PPROM, and abnormal amniotic fluid volume. Neither SPFR nor SEFR led to a change in the risk of gestational hypertension and placental abnormalities. In general, these comparisons mean that DET with fetal reduction may have a negative influence on the remaining fetal development and maintenance of pregnancies.

SET decreases the incidence of complications from multifetal pregnancies. In terms of success, DET increases the possibility of a live birth in one transfer cycle. Considering all available embryos, SET offers a better or similar overall pregnancy rate than DET [10, 29]. The SET success rate has been rising with the development of ART, and efforts have been made to establish SET as the standard of care [30]. Although SPFR and SEFR can help achieve a singleton pregnancy, SPFR is hardly predictable and SEFR requires additional surgical operation, which means that patients suffer more pain and a greater financial burden. Moreover, medical providers should be clear that many patients have difficulty confronting the prospect of fetal reduction and they may renege on prior agreements to undergo SEFR [31]. The expanded application of SEFR will also bring about some ethical controversies [32, 33]. This is also one reason why prospective randomized controlled studies are difficult to perform.

The strength of this study is that it’s the first comprehensive analysis of the clinical outcomes of singleton pregnancies derived from SET or DET twin pregnancies with subsequent fetal reduction based on a large sample size. Nonetheless, this study has the following limitations. First, a subgroup analysis of fetal reduction time was not performed because the fetal time was concentrated in a short time period. Second, threatened miscarriage, an important pregnancy complication, was missed due to deficiencies in our follow-up work. Third, a large proportion of cases in this study were followed up by telephone, and some important information was not very precise due to recall bias. Fourth, this is a single-center retrospective study, which prevents the generalizability of our findings.

## Conclusions

In summary, compared to an original singleton pregnancy, DET with subsequent fetal reduction by SEFR and SPFR was related to poor clinical outcomes. We recommend that DET with subsequent fetal reduction should only be considered as a rescue method for multiple pregnancy patients with potential complications, and SET is more advisable.

## Data Availability

The datasets used and/or analyzed during the current study are available from the corresponding author on reasonable request.

## Abbreviations

SET: Single embryo transfer
SEFR: Selective fetal reduction
SPFR: Spontaneous fetal reduction
DET: Double embryos transferred
OR: Odds ratio
CI: Confidence interval
GDM: Gestational diabetes mellitus
PPROM: Preterm premature rupture of membranes
ART: Assisted reproduction technology
IVF: In vitro fertilization
ICSI: Intracytoplasmic sperm injection
TVS: Trans-vaginal ultrasound; BMI: Body mass index
